# Navigating Quality in Lean Times: Sustaining Laboratory Standards Amidst Budget Constraints

**DOI:** 10.1111/ijlh.70117

**Published:** 2026-04-21

**Authors:** Gordon Sinclair

**Affiliations:** ^1^ London Metropolitan University London UK

## Abstract

Healthcare systems in wealthy countries are facing rising costs due to ageing populations and the prevalence of chronic illnesses, leading to constrained budgets. Pathology services, essential for accurate diagnosis and treatment, have merged to cope with these financial pressures. However, maintaining laboratory quality standards during economic downturns remains critical, as it directly affects patient outcomes, safety and satisfaction. This review explores evidence‐based approaches to maintaining quality standards amid financial difficulties. Major challenges include hiring freezes, heavier workloads and an increased risk of burnout, which is associated with higher error rates and staff turnover. The Conservation of Resources theory helps explain burnout by highlighting the importance of organisational support. Strategies to mitigate burnout include policies for digital disconnection, the promotion of transformational leadership among junior managers and the fostering of supportive leadership at higher levels. Additional measures involve sustaining competencies through Entrustable Professional Activities, structured feedback, cross‐training and Lean task prioritisation. Initiatives focused on staff well‐being, centred on autonomy, psychological empowerment and happiness, are crucial for preserving morale and performance. Overall, these strategies demonstrate that quality can be maintained through structured, evidence‐based interventions even during challenging times, thereby ensuring patient‐focused care and organisational resilience.

## 1 Introduction

In resource‐rich countries, healthcare costs are rising partly because of an ageing population and the associated increase in chronic diseases and comorbidities [1]. This leads to budgetary constraints on pathology; therefore, in the UK, pathology services have been consolidated to manage costs [2]. However, the current economic climate is also difficult and unlikely to improve [3]. Amid this increasingly challenging economic climate, maintaining quality remains essential, as it directly influences patient outcomes, treatment choices and ultimately, survival rates and quality of life [4]. In this review, quality will be defined as patient‐centred, staff compliance, staff competence and staff well‐being. A patient‐centred laboratory is accurately aligned with the needs, preferences and values of the patient population it serves. Patient‐centred care is essential for enhancing patient satisfaction, improving health outcomes and increasing adherence to treatment protocols [5]. Accurate and timely results are a key component of patient‐centred care. Deficiencies in staff adherence to established standard operating procedures can increase the incidence of medical errors and negatively impact patient outcomes, delay diagnosis and necessitate patient recalls [6]. Technical competence is similarly essential, particularly in any interpretive skills. Finally, staff well‐being, encompassing the psychological and physical health of healthcare professionals, can reduce *burnout* and increase job satisfaction and productivity [7]. Thus, this article aims to review evidence‐based strategies for sustaining quality under budget restrictions.

## 2 Challenges Under Budget Cuts

In the experience of the author, one of the first actions taken under budget restrictions is a freeze on recruitment, as this is seen as a softer option and avoids direct redundancies and severance costs [8]. At the same time, there is a relentless increase in clinical pathology workload [9]. A growing body of evidence demonstrates that *workload intensity* is one of the most significant predictors of occupational burnout among healthcare professionals [10]. Healthcare worker burnout is a substantial concern, characterised by emotional exhaustion, depersonalisation and a feeling of diminished personal accomplishment. Emotional exhaustion refers to feelings of being overextended and depleted of emotional and physical resources. In contrast, depersonalisation involves a sense of detachment from work or patients, which may lead to a negative attitude towards the job and the individuals being cared for. Diminished personal accomplishment reflects a low sense of competence and achievement at work [11]. Occupational burnout is directly correlated with errors [12], depression and anxiety, poor concentration and staff turnover due to early retirement or professional migration [13]. This is a significant risk to the laboratory. Increasing stress on the remaining staff is exactly the opposite of what is required.

The theory of Conservation of Resources (CoR) is a valuable model for understanding occupational burnout. CoR posits that individuals strive to acquire, retain and protect their resources, particularly in stressful environments. In the context of occupational burnout, this theory elucidates how the depletion of essential resources, emotional, physical or social, can precipitate exhaustion and disengagement among employees. This explains why workplace bullying results in burnout [14] as it erodes personal resources, leading to significant stress and burnout. Doğan et al. [10] suggest that the organisation plays a pivotal role in supporting staff and thus mitigating the risk of staff burnout. Their list of job resources comprises autonomy at work, initiative‐taking, job security, organisational justice and support from colleagues and superiors.

## 3 Organisational Mitigators of Burnout—Expectations

There must be a clear delineation between home life and work life, and a strict no‐contact policy outside work hours (to enforce the right to disconnect). Continuous work‐related communication (always on culture) disrupts individuals' personal lives. It makes it difficult for them to recharge, suggesting that promoting digital disconnection by setting boundaries can enhance mental health and overall productivity [15]. Having a clear organisational policy on digital disconnection is essential for safeguarding employees' physical and mental well‐being [15]. There is a legal component in the UK, as the Working Time Regulations 1998 stipulate that workers are entitled to a minimum of 11 continuous hours of rest every 24 h, along with a minimum rest period of 24 h per week or 48 h per fortnight (UK [16]).

## 4 Organisational Mitigators of Burnout—Junior Management Style

For centuries, the military has recognised the crucial role of junior leaders in sustaining troop morale and combat effectiveness [17]. A transformational leadership style is the single most significant influence on staff morale [18]. Transformational leadership comprises four key dimensions: idealised influence, which focuses on role‐modelling behaviours; inspirational motivation, which involves articulating goals and expectations; intellectual stimulation, which promotes knowledge sharing and creativity; and individualised consideration, which provides personalised coaching and mentorship [19].

Idealised Influence describes leaders who act as role models, demonstrating integrity and earning respect from followers. Such leaders inspire pride and admiration within their teams, fostering a culture built on trust and dedication [20]. Leaders who excel in this area can significantly improve employee performance by giving clear guidance and motivating team members towards shared goals [21].

Inspirational motivation occurs when a leader clearly communicates a compelling vision that motivates followers to work passionately towards common goals. Transformational leaders successfully share their vision and boost optimism within their teams, resulting in increased motivation and stronger engagement [22].

Intellectual Stimulation involves leaders fostering an environment that encourages creativity, problem‐solving and critical thinking among followers. Transformational leaders challenge the status quo, motivating team members to think beyond traditional boundaries and embrace innovative approaches [23].

Individualised consideration highlights the role of leaders in catering to the unique needs and growth objectives of their followers. Transformational leaders act as mentors, providing coaching and emotional backing to promote both personal and professional development [24]. This aspect of leadership fosters stronger relationships and increases employee satisfaction, as individuals feel valued and understood within their teams [25].

Healthcare scientists tend to be highly task‐oriented. We are good with things, less good with emotions. However, by embodying these traits, Junior leaders can inspire their teams to achieve exceptional outcomes while fostering an environment of growth, innovation and reducing occupational stress.

## 5 Organisational Mitigators of Burnout—Senior Management Style

Senior leaders who adopt a supportive leadership style can decrease occupational stress, burnout and staff turnover. When employees see their leaders as supportive and committed to their well‐being, their stress management significantly improves [26]. If the manager maintains a positive, upbeat attitude, they are more likely to motivate their team. Conversely, if their style involves giving negative feedback, it can lead to increased disillusionment and disengagement, both of which are components of occupational burnout.

## 6 Organisational Mitigators of Burnout—Organisational Support

When organisations create supportive environments that validate employees' roles and responsibilities, it enhances how employees understand the significance of their work [27]. Conversely, when organisational demands surpass support, employees may become overwhelmed, resulting in burnout due to a loss of purpose [28]. Practical management suggestions include increasing the frequency of regular feedback sessions, emphasising the importance of staff contributions and honouring individual achievements by including an achievement section in newsletters. Cross‐training staff helps redistribute tasks during shortages and prevents overloading key individuals. Through Lean prioritisation, identify non‐essential tasks and defer or reduce them during budget constraints to safeguard core quality processes. Repurpose the ‘huddles’ to share challenges and solutions, thereby reducing isolation. Redirect the Journal Clubs to include stress‐reducing activities.

## 7 Competency Maintenance

Defining competency levels in healthcare is essential for improving both the quality of care and staff morale. Competencies can be outlined through structured frameworks that specify expectations for different professional tasks, known as Entrustable Professional Activities (EPAs), and related milestones. These frameworks help clarify the competencies needed for unsupervised practise, fostering a shared understanding among staff of the standards required to attain and sustain their professional roles [29].

Gateway activities are essential for supporting development across these competency levels. They typically include opportunities for staff to demonstrate their skills in real‐world contexts, providing critical feedback for both individuals and the organisation. Participating in these activities not only helps staff advance in their professional growth but also enhances morale, as employees observe clear results from their efforts and see a pathway for future improvement [30].

Successfully navigating these frameworks enhances overall care quality, since staff who feel competent are more likely to engage positively with their roles and report higher job satisfaction and retention [31].

## 8 Staff Wellbeing

Agency in the workplace refers to employees' ability and freedom to make decisions within their roles. Ownership represents a personal sense of responsibility and connection to one's work. Both concepts are crucial for fostering a supportive environment that encourages professional development. Watkins et al. [32] highlight that professional autonomy is strongly linked to psychological empowerment, which can have a beneficial impact on job satisfaction and performance [32].

A positive work environment includes healthy relationships, management support and sufficient resources, but the discussion should not end there. We should aim to develop a work setting that promotes happiness. Happiness is described as an emotional evaluation of well‐being, which tends to focus on the positive rather than the negative [33].

Employee happiness reflects individuals' emotional reactions to their work [34]. However, for job satisfaction to reliably predict job performance, employees must also experience high levels of positive well‐being. This demonstrates the complex nature of the relationship; in fact, job satisfaction alone does not necessarily result in better performance without the influence of other psychological factors, such as positive well‐being [35].

## 9 Conclusion

Even with tight budgets, maintaining lab quality is achievable through intentional, evidence‐based approaches. Policies that focus on staff well‐being, set boundaries to prevent burnout and promote transformational leadership are crucial. Competency frameworks and cross‐training help uphold technical standards, while Lean prioritisation keeps essential processes effective. Ultimately, sustaining quality during lean periods is not only feasible but essential, as it ensures patient safety, clinical outcomes and staff retention. By applying these principles, laboratories can navigate economic challenges without compromising quality care.

## Author Contributions

Gordon Sinclair conceived the article, conducted the literature review, drafted and revised the manuscript and approved the final version for submission.

## Funding

The author has nothing to report.

## Ethics Statement

Ethical approval was not required for this study as it is a narrative review of previously published literature and did not involve human participants, patient data, or animal subjects.

## Consent

The author has nothing to report.

## Conflicts of Interest

The author declares no conflicts of interest.

## Data Availability

No new data were generated or analysed in support of this research. All data discussed are derived from previously published sources cited in the manuscript.
